# A homozygous variant disrupting the *PIGH* start‐codon is associated with developmental delay, epilepsy, and microcephaly

**DOI:** 10.1002/humu.23420

**Published:** 2018-03-30

**Authors:** Alistair T. Pagnamenta, Yoshiko Murakami, Consuelo Anzilotti, Hannah Titheradge, Adam J. Oates, Jenny Morton, The DDD Study, Taroh Kinoshita, Usha Kini, Jenny C. Taylor

**Affiliations:** ^1^ National Institute for Health Research Oxford Biomedical Research Centre, Wellcome Centre for Human Genetics University of Oxford Oxford Oxfordshire UK; ^2^ Yabumoto Department of Intractable Disease Research, Research Institute for Microbial Diseases Osaka University Osaka Japan; ^3^ World Premier International Immunology Frontier Research Center Osaka University Osaka Japan; ^4^ Weatherall Institute of Molecular Medicine, Radcliffe Department of Medicine John Radcliffe Hospital, University of Oxford Oxford UK; ^5^ West Midlands Regional Clinical Genetics Service and Birmingham Health Partners, Birmingham Women's and Children's NHS Foundation Trust Birmingham Women's Hospital, Mindelsohn Way Edgbaston Birmingham UK; ^6^ Radiology Department Birmingham Children's Hospital Birmingham UK; ^7^ Wellcome Trust Sanger Institute Wellcome Genome Campus Hinxton Cambridge UK; ^8^ Oxford Centre for Genomic Medicine Oxford University Hospitals NHS Foundation Trust Oxford UK

**Keywords:** developmental delay, exome, GPI‐anchor biogenesis, microcephaly, phosphatidylinositol N‐acetylglucosaminyltransferase, PIGH

## Abstract

Defective glycosylphosphatidylinositol (GPI)‐anchor biogenesis can cause a spectrum of predominantly neurological problems. For eight genes critical to this biological process, disease associations are not yet reported. Scanning exomes from 7,833 parent–child trios and 1,792 singletons from the DDD study for biallelic variants in this gene‐set uncovered a rare *PIGH* variant in a boy with epilepsy, microcephaly, and behavioral difficulties. Although only 2/2 reads harbored this c.1A > T transversion, the presence of ∼25 Mb autozygosity at this locus implied homozygosity, which was confirmed using Sanger sequencing. A similarly‐affected sister was also homozygous. FACS analysis of *PIGH*‐deficient CHO cells indicated that cDNAs with c.1A > T could not efficiently restore expression of GPI‐APs. Truncation of PIGH protein was consistent with the utilization of an in‐frame start‐site at codon 63. In summary, we describe siblings harboring a homozygous c.1A > T variant resulting in defective GPI‐anchor biogenesis and highlight the importance of exploring low‐coverage variants within autozygous regions.

Rare genetic variants that result in defective glycosylphosphatidylinositol (GPI)‐anchor biogenesis have been linked to a phenotypic spectrum of conditions that include learning disability, epilepsy, microcephaly, mild dysmorphic features, and other congenital malformations (Johnstone et al., 2017; Makrythanasis et al., 2016; Ng & Freeze, 2015). Although more than 20 genes have been implicated to date, analysis of exome data from 4,293 trios showed that disease causing variants in this pathway account for only around 0.15% of cases with developmental disorders (Pagnamenta et al., 2017). In this study, we interrogated the October 3, 2016 data release from the Deciphering Developmental Disorders (DDD) study (http://www.ddduk.org) comprising exomes for 7,833 parent–child trios and 1,792 singletons. We specifically searched for biallelic variants in the remaining eight genes critical to this pathway for which human disease associations are yet to be reported: *PIGB, PIGF, PIGH, PIGK, PIGS, PIGU, PIGX*, and *MPPE1*.

Patients were recruited to the DDD study under UK Research Ethics Committee approval (10/H0305/83; Cambridge South REC, and GEN/284/12; Republic of Ireland REC). In brief, reads were mapped to hs37d5 and variants were called using a combination of GATK, SAMtools, and Dindel for SNV and indels, and in‐house CoNVex for CNVs. Candidate de novo variants were called in trio data with DeNovoGear. More detailed methods have been described previously (Deciphering Developmental Disorders, 2017; Wright et al., 2015).

The only variant in the restricted set of eight GPI‐anchor biogenesis genes that passed all filter thresholds (<1% AF in all subpopulations from gnomAD) and fits with the presumed autosomal‐recessive inheritance pattern was an apparently homozygous c.1A > T; p.(Met1Leu) transversion in *PIGH* (MIM# 600154; NM_004569.3; rs761543313). This variant was identified in a boy (DECIPHER ID 265247 but referred to hereafter as IV‐1) with short stature, epilepsy, developmental delay, and behavioral difficulties. No other likely pathogenic variants have been identified in this boy. The c.1A > T variant was present in the gnomAD database (https://gnomad.broadinstitute.org) at an overall allele frequency of 0.002% (4/172732 or 4/25236 in the South Asian subgroup; all observations in the heterozygous state) but absent from the 1000 Genome Project (phase3v5b).

Unfortunately, exome coverage was very low at the *PIGH* start codon (2×, 3×, and 5× in the proband, mother, and father, respectively). Decreasing coverage below 15× increases the chance of heterozygous variants incorrectly being called homozygous, with very few heterozygous genotypes being called at 2× (Supp. Figure 1). However, the fact that both parents were heterozygous carriers of c.1A > T made the homozygous call in IV‐1 plausible. Review of high confidence SNVs on chr14 indicated that the c.A > T variant lay within a ∼25 Mb region of autozygosity between rs2275504 and rs10131300 (Supp. Figure 2). Together with the documented consanguinity (Figure 1A), this provided further support that the variant was homozygous. Bidirectional Sanger sequencing, performed using an ABI 3730XL instrument, later confirmed homozygosity in IV‐1 and his similarly affected sister (IV‐2), while both parents were heterozygous (Figure 1B). The variant was also detected in an RNA sample obtained from the mother, suggesting that the mutant transcript is not significantly degraded (Supp. Figure 3). In silico analysis shows the variant has a PHRED‐scaled CADD score of 27.6 and PolyPhen‐2 ranks it as possibly damaging.

**Figure 1:**
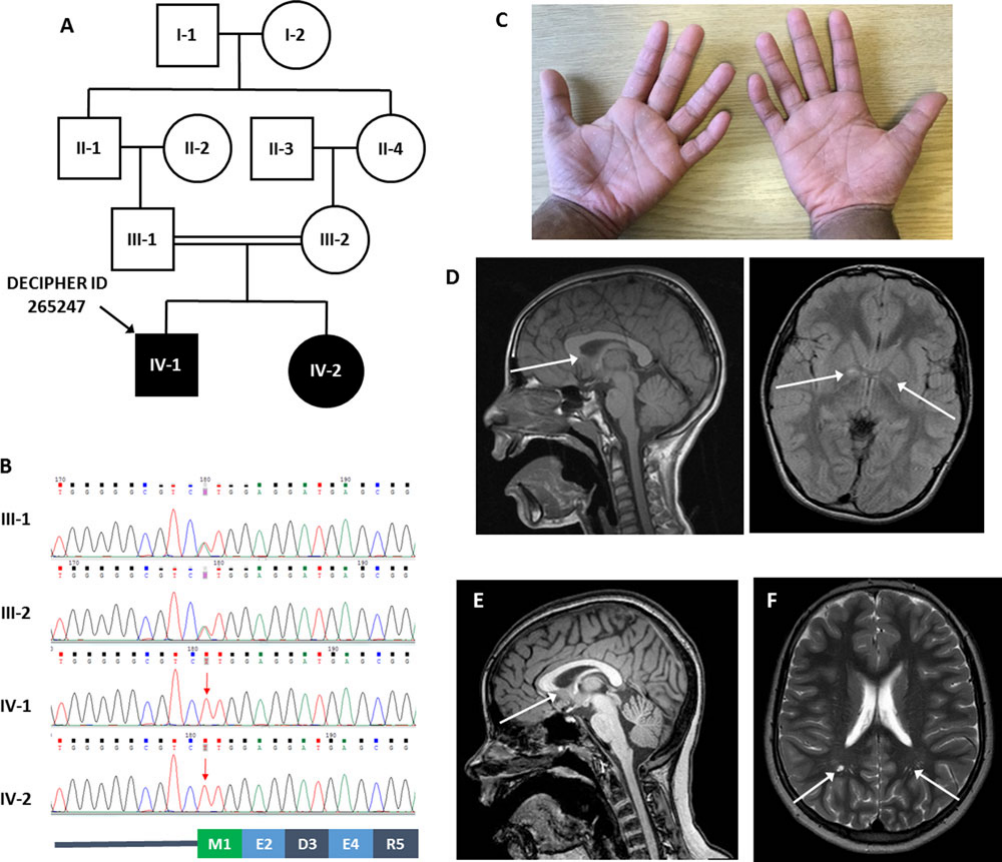
Pedigree, Sanger sequence data, and clinical images. **A**: Simplified pedigree for patient 265247, which shows parents to be first‐cousins. **B**: Genomic DNA was PCR amplified using FastStart Taq polymerase (Roche), with GC‐rich additive and primers CAGAGGGTGAAGAGTCCGTA and GGAAGAACGCACAACCATCA. Sanger sequencing of exoSAP purified PCR products, performed using BigDye v3.1 chemistry, confirmed that the c.1A > T variant was homozygous in both the proband and his affected sister (red arrows), while their parents were heterozygous carriers. The sequences shown are from the +ve strand primer but inverted to show the coding direction alongside the corresponding wild‐type amino acid sequence. The canonical methionine start codon is highlighted in green. **C**: Photograph of proband's hands demonstrating clinodactyly. **D**: Saggital T1 weighted MRI image of IV‐1 at 8 years of age demonstrating a dysplastic corpus callosum with a slightly truncated and rounded appearance of the rostrum of the corpus callosum. Axial T2 FLAIR image demonstrating abnormal signal within the globus pallidi bilaterally but more prominent on the right. **E**: T1 weighted MRI scan for IV‐2 showing a very slightly dysplastic corpus callosum, again with a truncated appearance of the rostrum. **F**: Axial T2 weighted MRI scan images for IV‐2 showing nonspecific increased high signal foci in the deep white matter bilaterally, most notably in the parietal regions

The proband (IV‐1) was born at 40 weeks weighing 3.12 kg (18th centile) to consanguineous Pakistani parents, following an uncomplicated pregnancy. His early development was reported to be normal; he walked at 14–15 months and had said his first words by 1 year of age. He developed febrile convulsions aged 14 months, which progressed to afebrile generalized myoclonic seizures at 3 years of age. These responded well to sodium valproate treatment. This treatment was stopped at the age of 12 years and he has remained seizure free until 18 years of age when he had one further seizure. Sodium valproate was subsequently recommenced. He had an episode of osteomyelitis of the left calcaneum at 6 years of age. He has chronic otitis media with effusion. He has mild learning difficulties and attended mainstream school with support. He also had behavioral difficulties, and as a child, he was described as being overfriendly and tactile with strangers. As a teenager, he struggled to make friends and could be aggressive.

On examination as a toddler, his height was on the 50th centile and weight over the 90th centile. At 9 years of age, his occipitofrontal circumference (OFC) was between the 0.4 and 2nd centiles. His growth rate decelerated during childhood, and by the age of 12 years 9 months, his height was 141.4 cm (7th centile), weight was 32 kg (6th centile), and OFC was 51.1 cm (1st centile). Clinodactyly of the 5th fingers (Figure 1C) and toes was noted. Neither parent has clinodactyly. He had forward turned ear lobes and creases on the posterior aspect of the helices. He had dry skin, which has been suspected to represent ichthyosis. He also has a speckled lentiginous nevus on the right side of his torso. A diagnosis of Smith‐Magenis syndrome was considered, but FISH for 17p11.2 was negative. Chromosome microarray analysis and *SCN1A* sequencing were normal. An MRI brain scan demonstrated subtle, non‐specific findings including small areas of abnormal signal in both globus palladi (right more than left) and a dysplastic rostrum of the corpus callosum (Figure 1D).

His younger sister (IV‐2) was born after a normal pregnancy at 40 weeks by normal vaginal delivery. She developed epilepsy with generalized tonic clonic seizures starting at 3.5 years of age, initially febrile and then afebrile. The seizures have proved difficult to control and surgical treatment has been discussed. Similar to her brother, her initial development was normal. She also initially attended mainstream school with extra help, but was later moved to a special school. She has difficulties with both verbal and nonverbal communication. She also has behavioral difficulties with temper tantrums and aggressive behavior toward strangers. Skin pigmentation is normal. She has 5th toe clindactyly, but no other dysmorphic features, pigmentary or skin abnormalities. Her growth parameters at the age of 7.5 years were: weight of 19.7 kg (2–9th centile), height of 121 cm (25–50th centile). OFC was 50.1 cm, putting her between the 0.4 and 2nd centiles, whereas 2 years previously her OFC had been between the 25 and 50th centiles. Similar to her brother, her MRI brain scan has shown a mildly dysplastic corpus callosum (Figure 1E) and non‐specific foci of abnormal high signal in the deep white matter (Figure 1F). A recent PET scan has shown increased uptake of glucose in the temporal regions. Neither parent has had similar symptoms or signs.

Abnormal levels of alkaline phosphatase in blood serum are a biomarker for some forms of GPI‐anchor deficiency, particularly when mutations involve *PIGV, PIGO*, *PGAP2*, or *PGAP3* (HPMRS1‐4). However, in this case, alkaline phosphatase was within the normal range for both siblings; most recent measurements were 107 IU/L (normal range 40–240) for IV‐1 and 228 IU/L (84–307) for IV‐2. Triglyceride levels were in the normal range for IV‐1 (1.20 mM) but significantly raised in IV‐2 (3.20 mM; normal range 0.45–1.62). Raised triglyceride levels have been associated with patients with PIGA deficiency and it is speculated that this is due to a secondary deficiency of GPIHBP1 (Tarailo‐Graovac et al., 2015).

One method commonly used to functionally assess variants in genes associated with the GPI‐anchor biogenesis is to perform FACS analysis on patient granulocytes. CD16 is often used as it is thought to be the most sensitive marker (Kato et al., 2014; Tanigawa et al., 2017). However, CD16 levels are also affected by how blood samples are handled and so patient samples are often compared to a small number of unrelated controls processed in the same batch. Such studies can potentially be confounded by the fact that granulocyte CD16 levels are variable among healthy individuals. In this study, FACS analysis demonstrated that IV‐1, IV‐2, and III‐2 all had median CD16 levels in between with the values obtained for two unrelated control subjects (Supp. Figure 4). To minimize the effects of genetic background and/or environmental factors, the functional consequences of the c.1A > T variant was assessed using plasmids containing wild‐type or mutant *PIGH* cDNA, which were transfected by electroporation (400 μl, 250 V, 950 μF, Gene Pulser II; Bio‐Rad, Tokyo, Japan) into *PIGH*‐deficient CHO cells. Co‐transfection with a luciferase‐expressing plasmid was used to monitor transfection efficiency. Two days after transfection, restoration of GPI‐AP was analyzed by FACS. Although no difference was observed when using a strong SR alpha promoter (pME), when expression was driven by a weaker TK promoter (pTK), mutant *PIGH* could not efficiently restore the surface expression of GPI‐APs (Figure 2A). Western blot analysis of protein expression using the pME construct demonstrated that the variant results in a truncated version of PIGH, consistent with utilization of an alternate in‐frame start site in exon 2 at the next available methionine codon at position 63 (Figure 2B).

**Figure 2:**
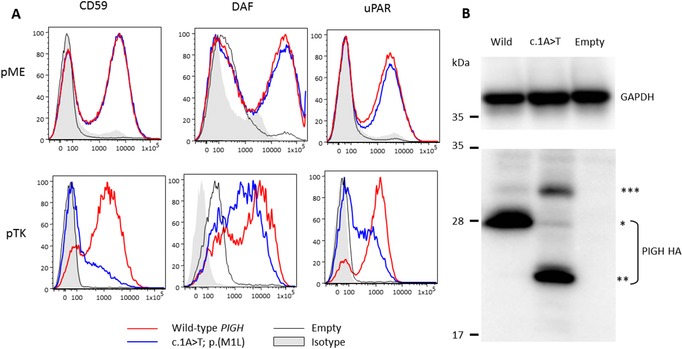
FACS and Western blot analysis using CHO cells. **A**: *PIGH‐*knockout CHO cells were generated and transfected with human wild‐type or c.1A > T mutant *PIGH* cDNA cloned into pME or pTK expression vectors. Restoration of the cell surface expression of CD59, DAF, and uPAR was evaluated by flow cytometry. Anti‐CD59 (5H8), DAF (IA10), and uPAR (5D6) were made in house. The mutant construct using the pTK promoter did not rescue surface expression of CD59 or uPAR as efficiently as the wild‐type construct, indicating that the variant results in reduced PIGH activity. **B**: Levels of expressed wild‐type and c.1A > T mutant HA‐tagged PIGH in pME‐vector transfected cells were analyzed by Western blotting using an anti‐HA antibody (C29F4 Rabbit mAb; Cell Signaling; 1:15,000). After normalization with luciferase activity and GAPDH (Anti‐GAPDH, AM4300 mouse mAb Ambion; 1:2,000), expression of full‐length PIGH (*) was reduced almost to zero and instead there was a smaller band (**), which likely represents a truncated version of PIGH in which translation starts from an alternative Met63 codon in exon 2. Although a larger band can be detected at around 30 kDa (***), it is unclear where this starts because there are no in‐frame methionine codons upstream

*PIGH* encodes a relatively small endoplasmic reticulum‐associated protein of 188 amino acids. Together with five other subunits, PIGH is an essential component of phosphatidylinositol (PI) N‐acetylglucosaminyltransferase, an enzyme that is critical for the first step in GPI‐anchor biosynthesis. The enzyme catalyzes the transfer of N‐acetylglucosamine (GlcNAc) from uridine 5′‐diphospho N‐acetyl glucosamine (UDP‐GlcNAc) to PI to form GlcNac‐PI (EC 2.4.1.198). Studies on the orthologous gene in yeast suggest the PIGH subunit is critical for biological activity (Yan, Westfall, & Orlean, 2001). Although truncation of amino acids 1–62 (the likely effect of the c.1A > T variant) represents a loss of 33% of the wild‐type PIGH protein, our results suggest that some residual activity is retained (Figure 2A). However, studies have shown that in certain situations, initiation from UUG codons can occur (Kearse & Wilusz, 2017) and so it is also possible a trace amount of full‐size PIGH, likely generated using Leu1 (Figure 2B), contributed to the residual activity.

Although a *PIGH* splicing variant in Belgian Blue cattle has been shown to result in arthrogryposis (Sartelet et al., 2015), the consequences of *PIGH* mutations in humans have not yet been described. In contrast, phenotypes associated with germline variants in the five genes encoding the other essential components of this complex have already been elucidated. Germline mutations in *PIGA* were originally reported to cause a X‐linked lethal disorder involving cleft palate, neonatal seizures, contractures, central nervous system structural malformations, and other anomalies (Johnston et al., 2012). Milder cases have since been described where the manifestation include profound learning disability, axial hypotonia, epilepsy, and hypsarrhythmia (Belet et al., 2014) or neurodegenerative encephalopathy with systemic iron overload and ichthyosiform skin lesions (Swoboda et al., 2014). A homozygous splice variant in *PIGQ* has been linked to Ohtahara syndrome (Martin et al., 2014) whilst a p.(R207*) alteration has been linked to intractable seizures, developmental delay, and optic atrophy (Alazami et al., 2015). A missense variant in *PIGY* resulted in a multi‐system disease involving dysmorphism, epilepsy, severe developmental delay, cataracts, and early death, while a regulatory variant was associated with a milder presentation limited to moderate development delay and microcephaly (Ilkovski et al., 2015). Mutations in *PIGC* are associated with epilepsy and intellectual disability (Edvardson et al., 2017), while those in *PIGP* have been linked to infantile epileptic encephalopathy (Johnstone et al., 2017). To sum up, although epilepsy is a common finding, many other variable features can co‐occur. We speculate that the relatively mild phenotype observed in the siblings described here is due to some residual activity and that biallelic *PIGH* mutations that result in zero activity would lead to a significantly more severe presentation. Further studies and the recruitment of additional patients with *PIGH* variants would be required to test this hypothesis.

GC content is well known to influence the resulting read coverage obtained by exome capture methodologies (Clark et al., 2011). Due to the presence of CpG islands at the 5′ ends of many genes, genetic variants in or around the start‐codon are therefore less likely to be called robustly than in other gene regions (Supp. Figure 5). In this case, we identified a homozygous c.1A > T with only 2× coverage. In a previous study, we identified a homozygous c.‐540G > A variant in the *PIGY* promoter, with only 4× coverage (Ilkovski et al., 2015). Variants with these levels of coverage are often considered low‐confidence and filtered out at early stages of analysis. Even if retained, low coverage variants are less likely to be genotyped accurately in terms of distinguishing heterozygosity from homozygosity (Supp. Figure 1). Together, these studies highlight the value of scrutinizing low coverage variants involving transcriptional or translational start sites, especially when they lie within regions of autozygosity. Our systematic analysis identified two siblings from the DDD study with homozygous disruption of the *PIGH* start codon, which leads to the use of an alternative start site and defective GPI‐anchor biogenesis resulting in a developmental disorder characterized by developmental delay, seizures, and microcephaly. Further cases are required to confirm the phenotypic range associated with rare biallelic *PIGH* variants.

## Supporting information


**Figure S1**. Percentage of variants detected at difference levels of coverage that are called homozygous in individual IV‐1. The numbers of variants called that fit into each coverage bin are indicated. Analysis was restricted to variants present in known databases such as dbSNP or COSMIC. Variants on the X and Y chromosomes were not included. Decreasing coverage increases the likelihood of a variant being called homozygous. Very few heterozygous genotypes are able to be called at 2x coverage, the level of coverage obtained at the site of the c.1A > T variant in *PIGH*.
**Figure S2**. B‐allele frequency plot for 2657 high confidence variants detected along chromosome 14. Only SNVs with a PASS flag and coverage of 15x or more are shown. *PIGH* (NM_004569.3) is situated at chr14:68,056,023‐68,067,017 and so appears to lie within a ∼25 Mb region of autozygosity between chr14:47,770,841‐72,939,495.
**Figure S3**. RNA analysis for individual III‐2. Blood was collected from the mother and two control subjects using PAXgene blood RNA tubes (Qiagen). RNA was extracted using the PAXgene Blood RNA Kit (Qiagen) and reverse‐transcribed using the QuantiTect RT kit (Qiagen). PCR amplification was performed using AGGTAGTGCTTTCTTTGCCTGA and CGAGACGACCAGGGCCGG primers where underlined base corresponds to the non‐reference base at rs7154825 which is the major allele. A) Gel image showing a PCR product of the expected size (403 bp) was obtained and no bands were observed in the RT negative control lanes. B) Sanger sequencing of PCR products was done using BigDye v3.1 chemistry on an ABI 3730XL instrument. For ease of comparison, the electropherograms are shown below the trace obtained using the mother's genomic DNA. The peaks corresponding to the wild‐type and c.1A > T alleles are of similar height, matching what is seen for genomic DNA. This suggests that the mutant RNA is not degraded significantly.
**Figure S4**. FACS analysis of CD16 expression in granulocytes from three family members and two healthy donor controls. Heparinized blood samples were treated with ACK lysis buffer and stained with a 1:100 dilution of anti‐human CD16 (3G8, Invitrogen). Samples were run on a BD FACSCanto and data analysed by FlowJo. Granulocytes were identified according to FSc and SSc profile. Median expression levels of CD16 for all three family members were in between two control subjects. All samples were collected at the same time and processed in the same way the following day.
**Figure S5**. Median number of no‐called samples in ExAC release 0.3.1 for A > T variants spaced at regular 100 bp intervals along all genes combined. Annotations were based on Gencode version 19 and using VEP version 85 as described at http://exac.broadinstitute.org. Values were taken for the “NCC” information field for all autosomal SNVs. Indels and variants where there were multiple non‐reference alleles were excluded.
