# Supplementary material for: A homozygous variant disrupting the PIGH start‐codon is associated with developmental delay, epilepsy, and microcephaly
Source: Hum Mutat. 2018 Mar 30;39(6):822–6. doi: 10.1002/humu.23420 (PMC6001798; doi:10.1002/humu.23420)
Supplement: Supplementary file 1 — Figure S1. Percentage of variants detected at difference levels of coverage that are called homozygous in individual IV‐1. The numbers of variants called that fit into each coverage bin are indicated. Analysis was restricted to variants present in known databases such as dbSNP or COSMIC. Variants on the X and Y chromosomes were not included. Decreasing coverage increases the likelihood of a variant being called homozygous. Very few heterozygous genotypes are able to be called at 2x coverage, the level of coverage obtained at the site of the c.1A > T variant in PIGH. Figure S2. B‐allele frequency plot for 2657 high confidence variants detected along chromosome 14. Only SNVs with a PASS flag and coverage of 15x or more are shown. PIGH (NM_004569.3) is situated at chr14:68,056,023‐68,067,017 and so appears to lie within a ∼25 Mb region of autozygosity between chr14:47,770,841‐72,939,495. Figure S3. RNA analysis for individual III‐2. Blood was collected from the mother and two control subjects using PAXgene blood RNA tubes (Qiagen). RNA was extracted using the PAXgene Blood RNA Kit (Qiagen) and reverse‐transcribed using the QuantiTect RT kit (Qiagen). PCR amplification was performed using AGGTAGTGCTTTCTTTGCCTGA and CGAGACGACCAGGGCCGG primers where underlined base corresponds to the non‐reference base at rs7154825 which is the major allele. A) Gel image showing a PCR product of the expected size (403 bp) was obtained and no bands were observed in the RT negative control lanes. B) Sanger sequencing of PCR products was done using BigDye v3.1 chemistry on an ABI 3730XL instrument. For ease of comparison, the electropherograms are shown below the trace obtained using the mother's genomic DNA. The peaks corresponding to the wild‐type and c.1A > T alleles are of similar height, matching what is seen for genomic DNA. This suggests that the mutant RNA is not degraded significantly. Figure S4. FACS analysis of CD16 expression in granulocytes from three family members and two heal [file HUMU-39-822-s001.pdf]

<u>Supplementary Information</u>

A homozygous variant disrupting the *PIGH* start-codon is associated with developmental delay, epilepsy and microcephaly

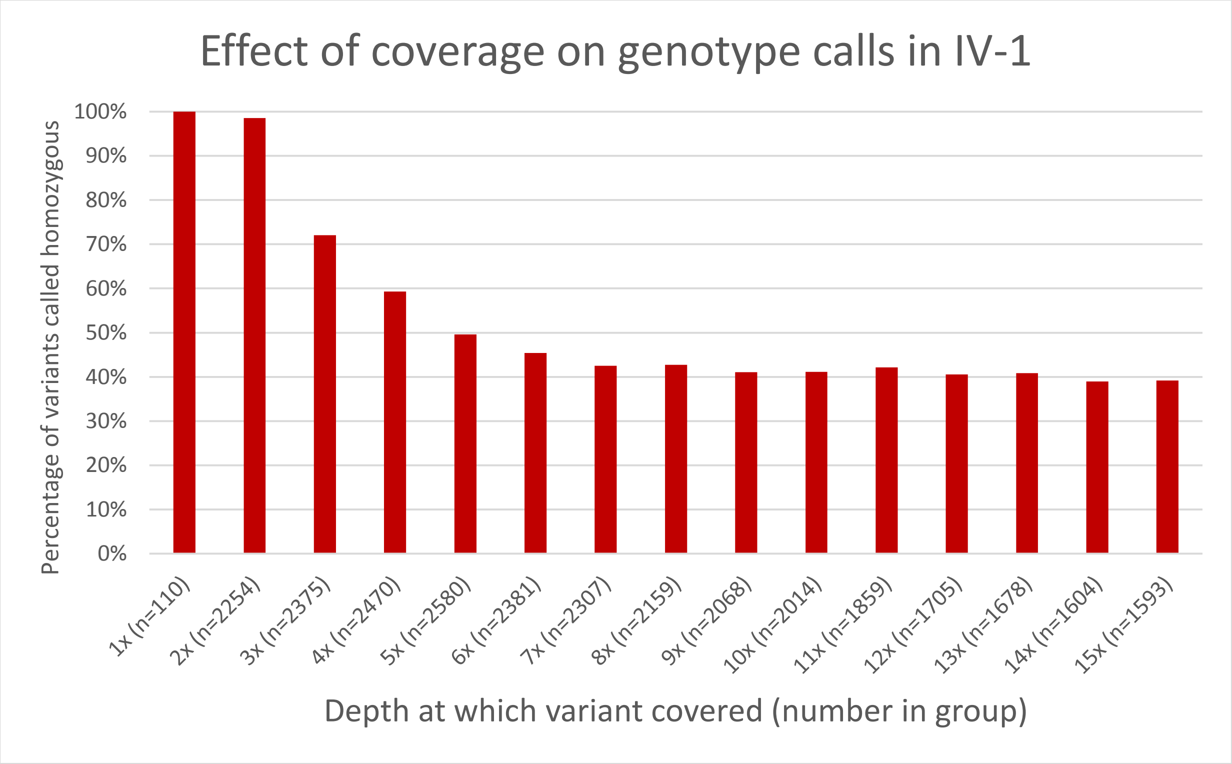

Figure S1. Percentage of variants detected at difference levels of coverage that are called homozygous in individual IV-1. The numbers of variants called that fit into each coverage bin are indicated. Analysis was restricted to variants present in known databases such as dbSNP or COSMIC. Variants on the X and Y chromosomes were not included. Decreasing coverage increases the likelihood of a variant being called homozygous. Very few heterozygous genotypes are able to be called at 2x coverage, the level of coverage obtained at the site of the c.1A>T variant in *PIGH*.

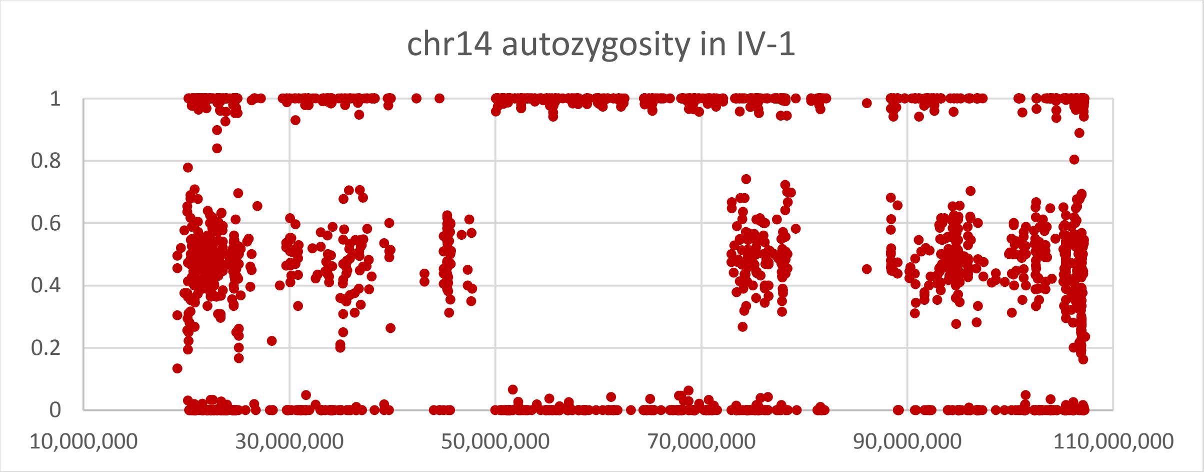

Figure S2. B-allele frequency plot for 2657 high confidence variants detected along chromosome 14. Only SNVs with a PASS flag and coverage of 15x or more are shown. *PIGH* (NM_004569.3) is situated at chr14:68,056,023-68,067,017 and so appears to lie within a ~25Mb region of autozygosity between chr14:47,770,841-72,939,495.

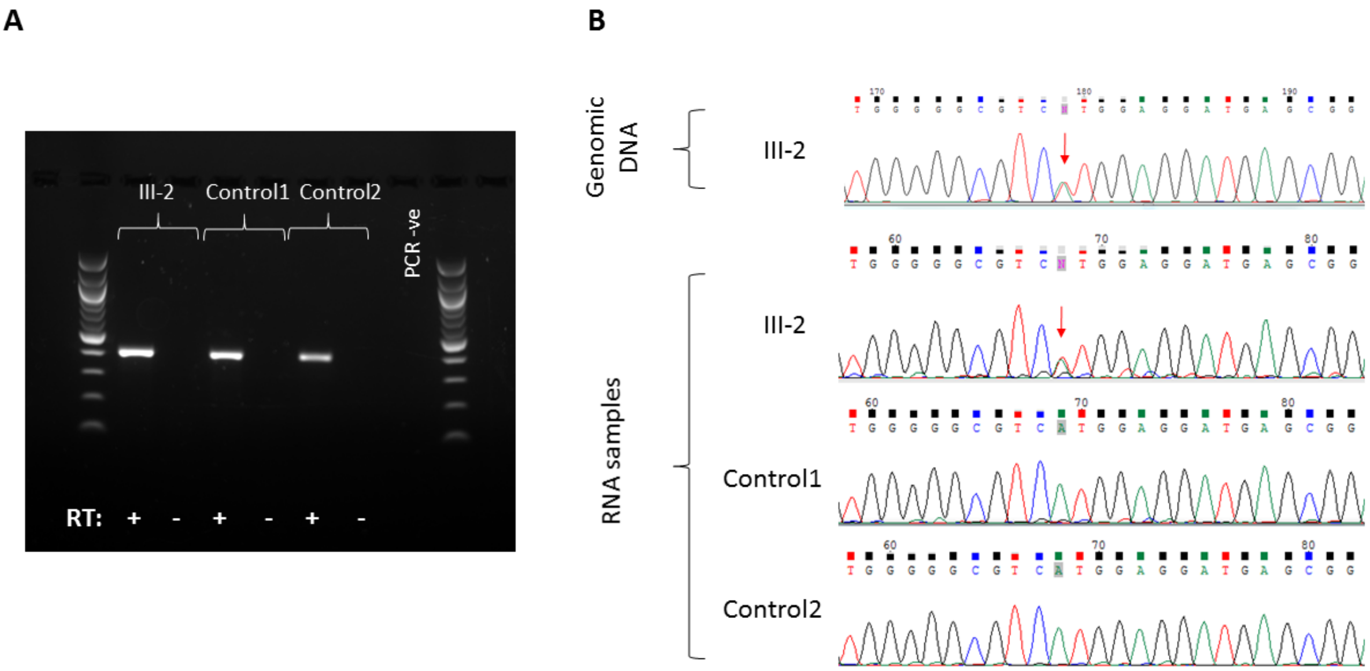

Figure S3. RNA analysis for individual III-2. Blood was collected from the mother and two control subjects using PAXgene blood RNA tubes (Qiagen). RNA was extracted using the PAXgene Blood RNA Kit (Qiagen) and reverse-transcribed using the QuantiTect RT kit (Qiagen). PCR amplification was performed using AGGTAGTGCTTTCTTTGCCTGA and CGAGACG<u>A</u>CCAGGGCCGG primers where underlined base corresponds to the non-reference base at rs7154825 which is the major allele. A) Gel image showing a PCR product of the expected size (403bp) was obtained and no bands were observed in the RT negative control lanes. B) Sanger sequencing of PCR products was done using BigDye v3.1 chemistry on an ABI 3730XL instrument. For ease of comparison, the electropherograms are shown below the trace obtained using the mother's genomic DNA. The peaks corresponding to the wild-type and c.1A>T alleles are of similar height, matching what is seen for genomic DNA. This suggests that the mutant RNA is not degraded significantly.

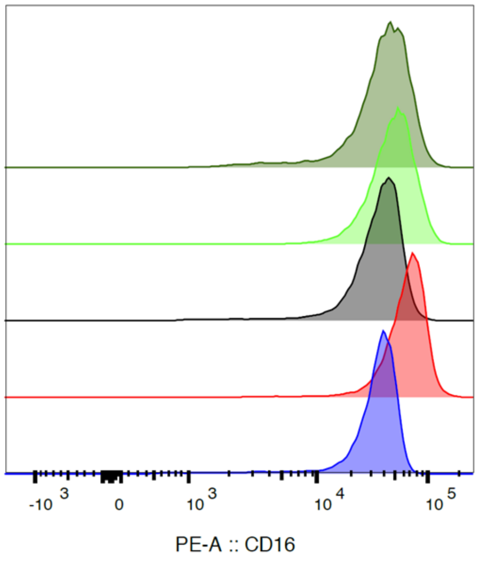

	Sample Name	Count	Median : PE-A
	**IV-2 (affected sister)**	13914	42138
	**IV-1 (proband)**	19771	49319
	**III-2 (carrier mother)**	15386	37801
	**Control 1**	26279	67668
	**Control 2**	21845	36547

Figure S4. FACS analysis of CD16 expression in granulocytes from three family members and two healthy donor controls. Heparinized blood samples were treated with ACK lysis buffer and stained with a 1:100 dilution of anti-human CD16 (3G8, Invitrogen). Samples were run on a BD FACSCanto and data analysed by FlowJo. Granulocytes were identified according to FSc and SSc profile. Median expression levels of CD16 for all three family members were in between two control subjects. All samples were collected at the same time and processed in the same way the following day.

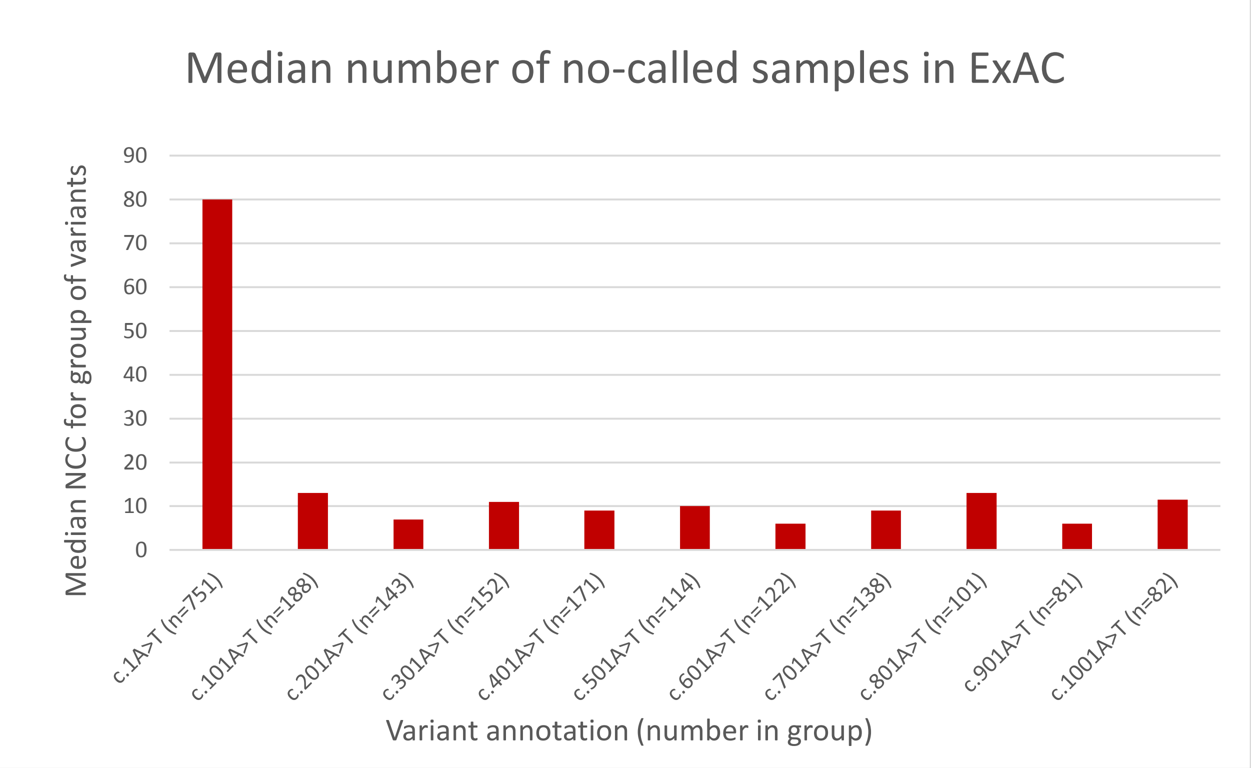

Figure S5. Median number of no-called samples in ExAC release 0.3.1 for A>T variants spaced at regular 100bp intervals along all genes combined. Annotations were based on Gencode version 19 and using VEP version 85 as described at http://exac.broadinstitute.org. Values were taken for the "NCC" information field for all autosomal SNVs. Indels and variants where there were multiple non-reference alleles were excluded.
